# Inpatient psychiatry care during coronavirus 2019 pandemic lockdown: Results from a department of psychiatry in northern Portugal

**DOI:** 10.1192/j.eurpsy.2021.828

**Published:** 2021-08-13

**Authors:** A. Vieira, F. Andrade, A.S. Machado, D. Barbosa, A. Sousa, I. Soares Da Costa, A. Silva

**Affiliations:** Psychiatry And Mental Health Department, Centro Hospitalar e Universitário de São João, Porto, Portugal

**Keywords:** COVID-19, inpatient care

## Abstract

**Introduction:**

COVID-19 pandemic and the consequent containment measures have a negative impact on mental health. Simultaneously, the fear of infection can discourage patients from seeking necessary care.

**Objectives:**

We aim to compare sociodemographic and clinical characteristics of inpatients admitted during the COVID-19 confinement period in Portugal vs. inpatients admitted in the same period the previous year.

**Methods:**

Retrospective observational study of inpatients admitted between March 19^th^ 2020 and May 1^st^ 2020 and the analog period of 2019 in a psychiatry inpatient unit of a tertiary hospital. Descriptive analysis of the results was performed using the SPSS software, version 26.0.

**Results:**

During the lockdown period, there were 30 admissions to the psychiatry inpatient unit, 55.2% less than the same period last year (n=67). The proportion of compulsory admissions and the average length of stay did not differ between the two periods. Regarding sociodemographic characteristics, in the confinement period inpatients were similar to the ones in the same period of 2019. In both periods, the majority of patients had previous psychiatric history (lockdown vs. same period last year: 95.5% and 90.0%) and a similar proportion of readmissions rate (previous year) was similar in the two groups (49.9% vs 47.6%). At discharge, the most frequent diagnostic groups were mood disorders (33.3% (n=10) and 34.3% (n=23)) and schizophrenia, schizotypal and delusional disorders (26,7% (n=8) and 31.3% (n=21)).

**Conclusions:**

Although there was an expressive reduction of admissions to the psychiatry inpatient unit during lockdown, the clinical characteristics of these patients were analogous to the same period in the previous year.

